# Private provision of health services in Georgia: a qualitative exploration of governance behaviours

**DOI:** 10.1136/bmjgh-2025-018922

**Published:** 2025-12-17

**Authors:** Mari Tvaliashvili, Mark Hellowell, Tomas Roubal, Akaki Zoidze, David Clarke

**Affiliations:** 1 World Health Organization Country Office for Georgia, Tbilisi, Georgia; 2 Global Health Policy Unit, Social Policy, School of Social and Political Science, University of Edinburgh, Edinburgh, UK; 3 School of Natural Sciences and Medicine, Ilia State University, Tbilisi, Georgia; 4 Governance, Law and Reforms, Performance, Finance and Delivery Department, World Health Organization, Geneva, Switzerland

**Keywords:** Delivery of Health Care, Universal Health Care, Health policy, Health systems, Health insurance

## Abstract

**Introduction:**

The private sector occupies a dominant position in Georgia’s health system, with most hospitals, primary care clinics, diagnostic facilities, pharmacies and insurance companies under for-profit ownership. Robust governance arrangements are required to align the profit-orientation of providers with health policy objectives.

**Methods:**

This paper examines governance arrangements in Georgia’s ‘mixed’ health system. It draws on document analysis, key informant interviews and a validation workshop. Analysis is guided by the WHO’s ‘governance behaviours’ framework, focusing on strategy, regulation, purchasing and information generation as well as mechanisms for policy dialogue, actor alignment and trust building.

**Results:**

Georgia has established a complex array of governance mechanisms for its dominant private health sector, but these remain weakly enforced. Strategic plans lack detailed implementation and budgetary integration; regulation and purchasing structures are fragmented; data systems and oversight capacity are limited; and consultation mechanisms underdeveloped—together constraining accountability, efficiency and progress towards universal health coverage.

**Concluding discussion:**

Georgia’s experience highlights a persistent gap between governance intent and implementation capacity. In highly marketised systems, sustained political commitment and investment in state capacity for enforcement, data use and stakeholder dialogue are essential to align private incentives with policy goals—and advance universal health coverage.

**WHAT IS ALREADY KNOWN ON THIS TOPIC**

- In many middle-income countries, the private sector provides a large share of health services. In such settings, weak governance undermines equity, financial protection and quality.
- Evidence on which governance mechanisms are applied, how they function and their effectiveness remains limited.
- Researchers have called for more in-depth qualitative studies that document how governance arrangements operate in specific contexts.

**WHAT THIS STUDY ADDS**

- This is the first qualitative assessment of governance arrangements for the private health sector in Georgia, where for-profit entities play a dominant role in service delivery.
- Strategy is emerging but remains under-resourced. Regulatory mechanisms are inconsistently enforced, and purchasing is non-strategic.
- Market concentration and vertical integration heighten risks of conflicts of interest, while weak data systems, monitoring and civil-society engagement limit transparency, accountability and trust.

**HOW THIS STUDY MIGHT AFFECT RESEARCH, PRACTICE OR POLICY**

- Strengthening governance requires consistent enforcement, strategic purchasing and investment in data and analytical capacity.
- Transparent, inclusive policymaking is needed to mitigate conflicts of interest that may arise, especially in vertically integrated markets.
- Further research is needed on how governments in resource-constrained settings can translate formal governance frameworks into practical impact, thereby advancing universal health coverage.

## Introduction

 The for-profit private sector plays an important role in the provision of healthcare in most middle-income countries (MICs).^1^ While proponents argue that the profit motive can stimulate efficiency and innovation, evidence suggests it may also encourage actions that undermine equity of access, financial protection and quality of care.^2^

To harness the potential benefits of for-profit ownership while mitigating the risks, governments and international agencies emphasise the importance of *governance*—defined by the WHO as ‘ensuring strategic policy frameworks exist and are combined with effective oversight, coalition-building, regulation, attention to system design and accountability’.^3^ This is a prerequisite for making sustainable progress towards universal health coverage (UHC).

The evidence base on governance of the private sector remains limited. A recent systematic review described the research on this topic as ‘patchy’ and of ‘varied quality’ and highlighted the need for more in-depth qualitative analysis, focusing on how governance is enacted in practice.^1^ This study responds to this gap: its aim is to provide a qualitative exploration of governance arrangements in Georgia—a country in which more than 80% of hospital beds, and the vast majority of primary care clinics, outpatient facilities, laboratories and pharmacies are owned by for-profit entities.^4^

To organise the analysis, we apply the WHO’s ‘governance behaviours’ (WHO-GB) framework.^5^ Building on earlier work by Travis and colleagues,^6^ this identifies six behaviours—developing strategy, enabling stakeholders, fostering relations, building understanding, aligning structures and nurturing trust—through which governments influence the private health sector. Our focus is on governance behaviours that relate to entities that have direct contact with patients and other consumers (thus, excluding regulation of manufacturers and distributors of medicines and medical equipment).

## Methods

### Theoretical framework

This study is guided by the WHO-GB framework (see definitions in table 1; and an outline of how these framed our questions in online supplemental annex 2). Unlike frameworks that conceptualise governance as a collection of static attributes (such as transparency, accountability, participation, integrity and capacity as in the ‘TAPIC’ framework of Greer *et al*),^7^ or that focus on value-based principles of ‘good governance’ (such as the rule of law and ethical norms as in the HSG framework of Siddiqi *et al*)^8^ the WHO-GB framework takes an operational, practice-oriented approach. As such, it provides a lens for analysing *how governance is enacted in practice*, which supports a structured assessment of strengths and weaknesses, and entry points for reform. Moreover, as this reflects WHO’s current conceptualisation of governance, use of this framework can enhance the relevance of findings for future technical cooperation, both in Georgia and other LMIC settings.^5^

**Table 1:** The six ‘governance behaviours’

	Governance behaviour	Definition
1	Deliver strategy	Government has articulated clear strategic goals and objectives for the health system and a clear definition of roles for the private health sector in achieving these.
2	Enable stakeholders	Government acts to influence the operation and performance of the private health sector through the use of: *regulations* (facility registration and licensing, and regulation of clinical practice, pharmacies, private health insurance, and economic regulation of the provider market); and *financing* (ie, purchasing and contracting).
3	Foster relations	The government has established inclusive policy processes, in which a broad range of stakeholders – including, but not limited to, the private health sector - plays an active role and with transparency.
4	Build understanding	Government has access to, and is able to act on, comprehensive, up-to-date and high-quality data on the operation and performance of the private health sector.
5	Align structures	Government has established organisational structures to achieve its strategic goals and objectives in relation to the private health sector.
6	Nurture trust	Government takes action to safeguard patients’ rights and broader welfare in their interactions with the private health sector.

### Study setting

As noted, most healthcare providers in Georgia are owned by for-profit entities.^4^ Under the *Hospital Development Master Plan* (2007–2012), most hospitals were transferred from the public to the private sector through tenders. The pharmaceutical and diagnostic sectors were privatised even earlier. The system is now dominated by a few vertically integrated corporate groups—such as PSP, Aversi and Georgia Health-Care Group—each owning hospitals, clinics and pharmacy chains.^4^

Public financing has expanded since the launch of the Universal Health-Care Program (UHCP) in 2013, which now covers more than 90% of the population. However, out-of-pocket payments remain high—reflecting gaps in service and cost coverage under the UHCP.^9^ The scheme is administered by the National Health Agency (NHA), while regulatory oversight is provided by the Regulation Agency for Medical and Pharmaceutical Activities (RAMPA), a body subordinated to the health ministry.

### Study design

We employed a qualitative design to: (1) examine how the WHO governance behaviours are enacted in practice; (2) identify their strengths and weaknesses and (3) explore entry points and priorities for reform. Data sources included key informant interviews (KIIs) and policy and regulatory documents. This triangulation of sources provided a comprehensive understanding of both formal governance arrangements and their implementation in practice. In addition, a validation workshop with selected respondents was held to corroborate and refine preliminary findings. Lists of (anonymised) key informants, workshop participants and documents are provided in supplementary materials (online supplemental annexes 1 and 2).

### Data collection

#### Key informant interviews

A semistructured interview guide was developed. This was informed by the WHO-GB framework and a related WHO guidance document, *Progression Pathway for Governance of Mixed Health Systems*, which includes a set of questions—broken down by governance behaviour, subdomain (eg, regulation and purchasing) and individual mechanisms (eg, facility registration and licensing)—intended to guide governance assessments.^5^ The interview guide is included in supplementary materials (online supplemental annex 3). Participants were identified through a combination of purposive and convenience sampling. A stakeholder mapping exercise, aligned with the six behaviours, guided the selection of key informants. Professional networks of the research team were used to facilitate contact with relevant individuals and institutions. Eligible participants included persons with experience in policymaking, or advocacy, regulation or purchasing as well as private sector owners and managers. No exclusion criteria were applied beyond unwillingness or inability to participate. Invitations were sent by email and followed up by telephone.

Between June and July 2024, a total of 20 KIIs were conducted. These comprised:

- Private sector facility owners/managers (n=6): representing various facility types (standalone vs networked), integration models (vertical vs horizontal), service domains (hospital vs primary care) and extent of UHCP engagement.
- Policymakers, regulators, policy experts (n=12): including health ministry staff, RAMPA and NHA staff, provider associations, a competition regulator and an independent consultant with substantial experience of working in health policy in Georgia.
- Financial sector stakeholders (n=2): specifically, a private health insurance (PHI) representative and an official from an international financial institution.

Interviews were conducted mainly in English and lasted approximately 60–90 min. The use of English was chosen due to the high proficiency of participants, who engage regularly with international partners, and by the language skills of the research team. However, a bilingual interpreter supported the interviews when participants preferred to respond in Georgian. Transcripts were subsequently checked for completeness and translation accuracy.

#### Ethical considerations

All participants received a plain-language information sheet and provided informed consent before participation. They were assured of anonymity to protect personal privacy and commercial or institutional interests. With consent, interviews were audio-recorded; however, in two cases, participants preferred that only written notes be taken. Patients and the public were not involved in the design, conduct, reporting of or dissemination plans for this research.

#### Document review

Documents were obtained from publicly accessible sources, including the websites of the health ministry, the Parliament of Georgia, RAMPA and international development partners (WHO, World Bank and United States Agency for International Development). Additional materials were provided by key informants or retrieved through targeted searches of government archives and media portals. The documents included national strategies, government decrees and resolutions, regulatory acts, policy reports and international partner assessments issued between 2013 and 2024—the period from implementation of the UHCP to the time of the research. Documents not directly addressing matters of system governance were excluded.

#### Validation workshop

To validate the findings, a workshop was conducted with a subset of key informants (n=10), including private sector managers, policymakers, regulators, policy experts and a representative of an international financial institution. Eight participants had previously been interviewed, and two were new to the study, having been unavailable during the interview phase.

During the workshop, preliminary findings were presented. Participants were invited to comment on the accuracy of data and interpretations and identify any gaps or misunderstandings. The session was facilitated by two members of the research team, who made notes on key points of consensus and disagreement.

Following the workshop, a written synthesis of draft findings, including those included/revised on the basis of workshop inputs, was circulated to all participants for further review. Six participants provided additional written feedback; and this was integrated into the final analysis.

#### Data analysis

Data from interviews, documents and the validation workshop were analysed using the Framework Method.^10^ Audio recordings were transcribed and checked for completeness, and field notes were expanded for consistency. Two researchers independently familiarised themselves with the material.

We used a predominantly deductive approach. An a priori analytical framework was specified in which the six WHO governance behaviours served as the top-level themes. For the *Enable Stakeholders* behaviour, covering regulation and purchasing, we defined second-level subdomains corresponding to these subdomains. Within these subdomains, we specified third-level mechanisms (specifically, within regulation, we considered each regulatory mechanism, and in purchasing, eligibility criteria, contractual specifications and payment models).

Assessment foci drawn from the *Progression Pathway for Governance of Mixed Health Systems* informed the operational definitions and boundaries of codes at the governance behaviour, subdomain and mechanism levels, as applicable (see online supplemental annex 3). The framework was applied to all sources. Data were indexed and charted manually in Microsoft Excel using a structured matrix reflecting this framework. During indexing and charting, limited inductive refinement was used to add or adjust codes where nuances were revealed in the data that were not captured by the pre-specified categories.

Coding definitions and interpretations were refined through team discussion and triangulation across interview, documentary and workshop data. Preliminary findings from the interview and documentary data were discussed at the validation workshop to test and refine interpretations, and subsequent written feedback from participants was integrated into the final synthesis.

## Results

The results are organised according to the six governance behaviours defined in the WHO-GB framework: deliver strategy, enable stakeholders, foster relations, build understanding, align structures and nurture trust. For each behaviour, we identify key strengths and weaknesses in governance arrangements, triangulating interview, workshop and documentary evidence. Quotations are used to illustrate recurring perceptions and experiences among participants. A synthesis of key findings is provided in table 2.

**Table 2:** A synthesis of key findings: strengths, weaknesses, challenges and illustrative insights for each governance behaviour

Governance behaviour	Main strengths	Main weaknesses/challenges	Illustrative quotations
Deliver strategy	Existence of a comprehensive *National Healthcare Strategy (2022–2030*) articulating goals for efficiency, cost containment and people-centred care.	Strategy is seen in the private sector as non-credible: it lacks detailed, costed, public domain implementation plans and integration in medium-term budgets. Uncertainty about government follow-through.	‘There is a lack of a long-term vision from the state.… It’s unclear how we should move forward.’ (Private Provider)
Enable stakeholders	**Regulation:** Progressive updates to licensing standards; piloting of selective contracting;	**Regulation:** Weak and inconsistently enforced; outdated standards; limited clinical governance.	**Regulation:** ‘The documents are unclear… when we suggest working together [with RAMPA])to clarify them, we don't see any willingness.’ (Private Provider)
	**Purchasing:** payment reform (prohibition of ‘balance’ and ‘extra’ billing and caps on co-payments).	**Purchasing:** limited strategic purchasing; performance levers applied inconsistently.	**Purchasing:** ‘Selective contracting… [is])something they are considering, but the process is not very active at the moment’ (Policy Stakeholder)
Foster relations	Formal legal requirements for consultation with non-state actors; private sector engaged in some policy processes (eg, diagnosis-related group (DRG) reform).	Consultation perceived as directive, not deliberative; key decisions finalised before input; no institutionalised mechanisms for patient or community participation.	‘During the meetings, the health ministry typically presents the changes they have made or are about to make’. (Private Provider)
Build understanding	Mandatory reporting to national health information system (HIS); growing government recognition of data’s value; early electronic tools in place.	Variable compliance; fragmented reporting; limited data scope (focused on claims, not quality); weak analytical capacity in government; no provider feedback or public access to data.	‘We could make adjustments… but for these tools to function properly, they need extra investment’. (Government Official)
Align structures	Private sector well-integrated in essential services delivery. Regulatory framework for gatekeeping and non-communicable diseases (NCD) care pathways; formal recognition of primary care as central.	Weak enforcement and underfunding erode alignment between private provision and primary care goals; stagnant capitation rates; private primary care providers shifting toward out-of-pocket (OOP)-funded services.	‘It is these additional (non-UHCP)services that allow us to survive’. (Private Provider)
Nurture trust	New restrictions on co-payments under UHCP enhance transparency—offering protection for patients against financial exploitation.	Limited scope of protections; weak enforcement of patient rights; lack of redress mechanisms and social accountability; underdeveloped patient organisations.	‘Patient associations are not very well developed in Georgia… In most areas, they are less developed’. (Policy Stakeholder)

### Deliver strategy

The *National Healthcare Strategy 2022–2030* provides the overarching framework for reform.^11^ This emphasises cost containment, efficiency and development of a more primary care-oriented service delivery model. The key reforms include stricter facility licensing requirements; expansion of selective purchasing within the UHCP; enhancement in the role and capacity of primary care; greater aggregation in diagnosis-related group (DRG) payments and prohibition of ‘extra’ and ‘balance’ billing for UHCP-funded services. Collectively, these reforms mark a clear shift away from the previous policy stance, which—in effect if not by design—encouraged the expansion of specialised care, much of it paid for directly by patients, resulting in a hospital-centric, fragmented and commercialised service delivery network.^12 13^

Key informants consistently welcomed the *Strategy* as a step towards greater policy coherence and direction. However, several questioned its feasibility given the scale of change envisaged and the absence of detailed implementation plans. Private providers, in particular, expressed uncertainty about whether some policy commitments—such as strengthening of primary care—would be backed by adequate, predictable financing. Participants noted that reforms will only be credible if they are fully costed and integrated into medium-term expenditure frameworks. Without this, the *Strategy* remains an aspirational document, offering limited guidance to market players on how to plan, invest and allocate resources. As one manager of a private health facility observed:

We continue to feel there is a lack of a long-term vision from the state. As a result, we don’t know where we’re headed, or how to develop our business, or even if this business is going to be needed. It’s unclear how we should move forward. (Private Provider)

### Enable stakeholders

This governance behaviour is concerned with how governments use regulatory and financial instruments to shape private providers’ structure, conduct and performance in alignment with policy goals. Because of its breadth, we present the findings in two subdomains, regulation and purchasing; and, within the former, by specific mechanisms (namely: facility licensing, regulation of clinical practice, regulation of retail pharmacy, regulation of PHI; and regulation of competition/market structure).

#### Facility registration and licensing

Responsibility for licensing lies with the RAMPA, which sets standards, conducts inspections and applies sanctions for non-compliance. For many years, licensing requirements were limited; more recently, they have been strengthened in some clinical settings—for example, there have been updates to infection prevention and control standards in hospitals. Several informants identified primary care as an area of concern, with current standards viewed as insufficient to safeguard quality of care.

Private providers reported persistent ambiguity in the wording of regulations as well as inconsistency in their enforcement.

Yes, there is a whole suite of regulatory documents - but the problem is that I can interpret these one way, and they can interpret them in another. The documents are unclear, and when we suggest working together [with RAMPA] to clarify them, we don't see any willingness on their part to do so. (Provider)

Penalties were perceived as insufficiently risk-based, with large fines levied for infractions that posed little material risk to patient safety—creating business uncertainty and straining relations with the regulator.

#### Regulation of clinical practice

All providers—public and private—must follow national treatment standards and clinical protocols when delivering UHCP services. A National Council—with representatives from the health ministry, RAMPA, providers and professional associations—develops and updates these mandatory instruments, alongside advisory clinical guidelines. In practice, such instruments cover only a minority of conditions, and there is no routine schedule for updates.

Several respondents highlighted insufficient safeguards against conflicts of interest: there is no transparent evidence-review process for instrument updates, and association representatives are not required to disclose commercial links (eg, with pharmaceutical companies), raising concerns about the potential for bias in standards, protocols and guidelines.

Enforcement was widely described as irregular and under-resourced—hampered by unclear indicators, weak monitoring frameworks and limited technical capacity within RAMPA and the NHA. As one key informant noted:

The health sector requires regulation, but it must be adequately designed and supported by clearly defined and consistently applied monitoring mechanisms. The nature of audits and their interpretations should be standardized, as inconsistencies in regulatory interpretation among individuals pose a significant challenge. (Government Official)

#### Regulation of retail pharmacies

The Law on Medicines and Pharmaceutical Activities sets standards for pharmaceutical quality, safety and pricing.^14^ Enforcement remains problematic, including in areas of public health importance: for example, regulations exist on the prescription and sale of antimicrobials for human use, but key informants reported that antibiotics can, in practice, be purchased over the counter without prescription.

Despite external reference pricing, medicine prices remain high relative to international benchmarks. Out-of-pocket (OOP) spending on medicines is the leading driver of catastrophic health expenditure—accounting for nearly 90% of OOP payments among the poorest households with catastrophic costs in 2018.^15^

The retail pharmacy market is highly concentrated: the three largest chains own about one-third of outlets. Several chains are vertically integrated—owned by companies that also hold pharmaceutical companies, hospitals and insurers.^16^ Combined with weak enforcement, such integration generates conflicts of interest and incentives for irrational prescribing.^17^

A 2021 assessment by the Georgian Competition and Consumer Agency found that physicians frequently indicated brand names in prescriptions, reinforcing overuse of branded rather than generic medicines.^17^ Despite a 2022 reform requiring prescribing by International Non-Proprietary Name, brand promotion remains widespread.

Doctors who are paid relatively low salaries, such as 300 or 500 Georgian lari a month [around US$ 100], may be more susceptible to prescribing pharmaceutical products that align with the interests of [their] owners. This creates a potential conflict of interest, where financial incentives may drive prescribing decisions, undermining ethical standards and patient care. (Government Official)

#### Regulation of PHI

PHI accounted for 7% of total health expenditure in 2023^18^ and covered about 19% of the population.^18 19^ PHI coverage is mainly held by people on high incomes as well as people in certain categories, such as military personnel.^19^ Most policies are employer based; individual plans are scarce, expensive and offered by few insurers. Key informants—including insurance industry regulators—highlighted affordability and limited consumer choice as key challenges:

If you’ re currently employed, you may be able to afford a private insurance package, but as an individual, it is expensive and also difficult to obtain. Most [insured] people have employer insurance. With individual coverage, you don’t receive full coverage, and you may experience longer waiting periods. For self-employed individuals, it is especially challenging. Only three insurance companies offer individual packages. (Policy stakeholder)

UHCP eligibility rules prohibit dual public–private coverage (except for specified groups), constraining the development of complementary PHI to cover copayments or services excluded from the public package. Otherwise, the regulations focus, rather narrowly, on solvency and consumer protection, with no requirements on minimum benefits, pricing or standardisation. One key informant highlighted the resulting risks: underinsurance and mis-selling, risk segmentation (via exclusions and waiting periods) and adverse selection, which may lead to premium increases, narrower service coverage and higher cost-sharing.

#### Regulation of competition/market structure

Service delivery in Georgia is highly fragmented: over 239 hospitals operate nationwide, averaging 63 beds per facility compared with a European average of 297.^12 16^ The proliferation of small hospitals increases fixed costs, reduces quality of care (eg, due to the lack of training opportunities for staff), and complicates regulatory oversight.

However, fragmentation in service delivery coexists with vertical (and in some cases horizontal) integration. As noted above, many larger chains are vertically integrated across pharmaceuticals, insurance and health services, leading to concentration of market power.^20^

There are no sector-specific rules to monitor, limit or unwind such integration, and the *Strategy 2022–2030* proposes no new measures in this area.^17^

Large companies and holdings are both vertically and horizontally integrated, meaning they control almost every aspect of the market. They don’t need to pay others because pharmacists are their employees, allowing them to dictate which medicines are sold. They have the power to control the market. On the other hand, small companies face significant challenges. In some cases, they resort to unethical behaviour, such as paying doctors to prescribe specific medicines. (Policy Stakeholder)

#### Purchasing

Public financing is the dominant source of revenue for private providers: among the five largest corporate groups, the share of income from the UHCP and other public sources ranged from 33% (New Hospitals) to 70% (Evex Clinics) in 2022.^21^

Despite this, the NHA operates more as a passive payer than a strategic purchaser. Barriers to UHCP entry are generally low. Providers do not hold individualised contracts; instead, requirements are defined in regulation, leaving little room for negotiation over service volumes, quality indicators or performance targets. The NHA’s monitoring is largely restricted to claims assessment. Patients are free to choose their provider, while providers retain discretion over whom to admit or enrol.

All contracts are regulated by ministerial decree. The UHCP is governed by a decree. The contract between the provider and the NHA is based on this decree. If I am a provider and wish to participate in the program, and I meet all the requirements outlined in the governmental decree, I simply submit a letter or application. (Government Official)

The government has made some attempt to use eligibility criteria to influence network structure. To qualify for UHCP financing, urban primary care providers must employ at least five family doctors and nurses and enrol a minimum of 13 000 people, encouraging consolidation into larger group practices.^19^ International accreditation is being made compulsory for all facilities receiving UHCP funds. Selective contracting has been piloted in maternity care, and Strategy 2022–2030 envisages its gradual extension to specialist outpatient services.

However, key informants noted the sizeable political risks of a wider expansion of selective contracting—and suggested that the necessary level of political commitment is lacking.

Selective contracting cannot work unless a clear decision is made. It’s up to the authorities to think through and decide how to structure the entire system. It’s something they are considering, but the process is not very active at the moment - it’s still just an idea in the air. (Policy Stakeholder)

Progress is most evident on payment reform. In 2022, fee-for-service payments for hospitals were replaced by DRG-based tariffs. As noted above, the *Strategy 2022–2030* also envisages DRG consolidation into fewer, broader groups, a ban on ‘extra’ and ‘balance’ billing, and caps on copayments—steps towards a more transparent, cost-reflective purchasing model. While these changes may improve efficiency and financial protection, they may also, key informants suggest, create risks of patient selection or ‘cream-skimming’.^2^ For example, one stakeholder reported that hospitals are focusing on wealthier patients able to ‘self-pay’ for uncovered services; while some are refusing to admit the sickest patients—although, the government is acting to discourage this.

Using DRG, providers can engage in cherry-picking patients and selectively choosing who to admit. A new regulation has been introduced where, if a provider has available capacity but refuses to admit a patient from an ambulance, they will not receive patients from ambulances for the next seven days. (Private Provider)

### Foster relations

Legislation formally requires the government to consult non-state actors in the formulation of health policy. Government officials highlighted effective private sector interactions in developing the Strategy 2022–2030, and in (ongoing) DRG reform, as examples of collaboration. However, many participants reported that policy decisions were often finalised before consultation occurred. Private-sector representatives described these interactions as largely *directive rather than deliberative*—with meetings focused on communicating government decisions rather than genuine policy dialogue.

Regarding DRG reform, there is a council established that includes representatives from various institutions such as the Ministry of Finance, Parliament, the health ministry, and provider associations. However, this council is not very effective. During the meetings, the health ministry typically presents the changes they have made - or are about to make. (Private Provider Association Representative)

Some participants also noted the lack of institutionalised mechanisms for social participation (which could form part of efforts to enhance transparency and accountability): patient associations and other civil society organisations are generally weak in Georgia and seldom engaged in policy development or monitoring.^22^

### Build understanding

Reporting into the national HIS is mandatory for all licensed facilities, regardless of ownership. While compliance is uneven, government officials noted ongoing efforts to improve reporting consistency and quality. Persistent challenges include, however, duplication of entries, non-standardised data collection processes and variable data quality, all of which limit the system’s usefulness for planning and decision-making.

The scope of data collection is also narrow—focused primarily on enabling the NHA to process UHCP claims and capitation payments, rather than monitoring quality of care or clinical outcomes. Key informants observed that the HIS has significant potential to support data-driven analysis and performance management, but this remains unrealised due to weak data quality, limited scope and insufficient capacity within public agencies.

Based on analysis of electronic data, we could make adjustments and corrections in the system. But for these tools to function properly, and be used properly, we need extra government investment and resources. (Government Official)

Participants also highlighted that providers receive little feedback from the state, constraining quality improvement efforts. Patients likewise lack access to comparative information—such as mortality or readmission rates—that could potentially lead to more informed patient choices and provider accountability.

### Align structures

The private sector plays a central role in delivering both essential public health services, including immunisation, screening and management of common NCDs. Recent regulations strengthen the gatekeeping function of primary care providers and define clinical pathways for four major NCDs—guiding referrals to specialist outpatient and hospital services. These measures aim to reduce self-referral and improve care coordination;^23^ however, their impact remains uncertain due to weak enforcement, limited monitoring and a lack of financial incentives for compliance.

Chronic underfunding constrains the scope and reach of primary care—despite long-standing policy intentions, reiterated in the *Strategy 2022–2030*, to strengthen this domain. UHCP capitation rates have been fixed in cash terms since 2013, significantly eroding their real value—by an estimated 35% by 2021.^24^ Interviews indicate that many private providers have responded by scaling back UHCP participation and expanding self-pay services to remain viable.

We are surviving largely due to co-payments and out-of-pocket payments from patients. It is these additional services that allow us to survive. (Private Provider)

Together, underfunding and weak incentives and monitoring create a risk that patients will be diverted from publicly funded primary care to ‘self-pay’ services, undermining continuity and weakening alignment with the intended model of care.

### Nurture trust

Some regulations—such as the post-2022 restrictions on copayments—offer a degree of financial protection for patients, but these apply only to UHCP-financed services. Beyond these, informants described weak and inconsistently enforced safeguards, alongside a lack of institutionalised social-accountability mechanisms.^25^

In practice, price transparency is limited (itemised bills and advance quotations are not routine). Effective redress is hard to obtain: complaints pathways are fragmented across state agencies, providers and insurers, with unclear timelines and limited feedback to patients. Several respondents pointed to low public awareness of entitlements and complaints procedures and to under-resourced patient organisations. These gaps undermine trust, particularly where patients face unexpected charges or disputed clinical decisions and interact with issues described above (eg, conflicts of interest), creating a perception that patients’ rights are at risk.

Patient associations are not very well developed in Georgia. In mental health we have patient associations, and in cancer as well. But in other areas, they are less developed. When such associations are created and active, patients can be involved [in policymaking]. (*Policy Stakeholder*)

## Discussion

This study applied the WHO-GB framework to examine how Georgia steers a predominantly private health sector. Institutional arrangements and policy intent are clearly articulated, but implementation is uneven. The *Strategy 2022–2030* offers a coherent roadmap focused on efficiency, cost containment and stronger primary care; yet credibility is weakened by the absence of detailed implementation plans, transparent costing and integration with medium-term budgets.

Across governance functions, formal mechanisms exist but are weakly operationalised. Licensing standards and facility inspections have improved on paper but are inconsistently enforced; clinical practice instruments cover only a minority of conditions and are irregularly updated and monitored; and ownership concentration with vertical integration across hospitals, pharmacies and insurers creates conflicts of interest that remain largely unaddressed. Payment and purchasing reforms—DRG-based tariffs, mandatory accreditation and selective-contracting pilots—represent progress, but the NHA still acts mainly as a passive payer. Without individualised contracts, explicit performance requirements and active monitoring, the state’s purchasing power is underused.

The health information system remains focused on claims administration rather than quality or outcomes, and limited analytical capacity within the NHA and other agencies constrains data-driven decision-making. Georgia’s high degree of corporate integration amplifies risks of conflicts of interest and regulatory capture, while countervailing forces—civil-society organisations and patient groups—are under-resourced and seldom engaged. Strengthening their presence and role in policy dialogue would bolster transparency and accountability.

These patterns mirror experiences in several middle-income countries where rules are clear but enforcement capacity is constrained (eg, Armenia, Kyrgyzstan),^26^,^28^ and where public authorities’ ability to govern is limited when large parts of the sector are owned by well-connected commercial interests.^29^,^32^

By contrast, Estonia and Thailand show how sustained investment in state capability and digital infrastructure enables governments to use contracting, accreditation and interoperable data to align private-sector incentives with public goals.^30 31^ These examples show that governance can be effective where there is political commitment to strengthening it can be established and sustained.

For Georgia, four priorities emerge for strengthening governance of the private health sector. First, translate the Strategy 2022–2030 into an actionable plan aligned with medium-term budgeting, transparent costing and measurable performance targets. Second, reinforce regulatory coherence and enforcement by updating licensing and clinical standards, adopting risk-based inspection that emphasises support and compliance, and extending regulation to address conflicts of interest and market power. Third, make purchasing genuinely strategic by expanding selective contracting based on population need, introducing individual provider contracts with clear access and quality requirements, and—critically—strengthening the NHA’s analytical capacity to link payment to performance. Finally, deepen participation and transparency by institutionalising government–provider–civil-society dialogue, strengthening patient-rights legislation and complaints/redress mechanisms, and expanding public reporting of quality indicators through the HIS.

Together, these measures would strengthen the incentive-and-accountability environment within which the private health sector operates in Georgia—reinforcing the stimulus to quality and efficiency, rebuilding trust between the state and private providers and bringing private sector actions into alignment with public goals.

This study is the first to apply the WHO Governance Behaviours framework to a ‘real-world’ mixed health system. It provides a detailed account of how governance mechanisms function in practice, triangulating perspectives from policymakers, regulators and private providers. However, several limitations should be noted. The study could not include civil society or patient organisations, whose perspectives would—for reasons noted above—have enriched the analysis. Most interviewees were senior officials or corporate representatives, and while their insights are essential for understanding governance, they may not capture implementation realities at the clinical level. As with all qualitative research, findings may reflect respondent biases despite triangulation with policy documents. Future comparative studies should examine how similar governance challenges manifest across countries with different institutional capacities and degrees of market dominance.

## Supplementary material

10.1136/bmjgh-2025-018922 online supplemental file 1

## Data Availability

No data are available.

## References

[1] Goodman C, Witter S, Hellowell M (2024). Approaches, enablers and barriers to govern the private sector in health in low- and middle-income countries: a scoping review. BMJ Glob Health.

[2] Pita Barros P, Fifteen SLC, Pauly MV, Mcguire TG, Barros PP (2011). Handbook of Health Economics.

[3] World Health Organization (2007). Everybody’s business: strengthening health systems to improve health outcomes: who’s framework for action.

[4] Richardson E, Berdzuli N (2017). Georgia: Health System Review. Health Systems in Transition.

[5] World Health Organization (2025). The progression pathway for governance of mixed health systems. https://ccpsh.org/research/progression-pathway-governance-mixed-health-systems.

[6] World Health Organization (2020). WHO advisory group on the governance of the private sector for universal health coverage. engaging the private health service delivery sector through governance in mixed health systems. https://www.who.int/publications/i/item/9789240018327.

[7] Greer SL, Wisma M, Figueras J (2016). Strengthening health system governance: better policies, stronger performance. https://eurohealthobservatory.who.int/publications/m/strengthening-health-system-governance-better-policies-stronger-performance.

[8] Siddiqi S, Masud TI, Nishtar S (2009). Framework for assessing governance of the health system in developing countries: gateway to good governance. Health Policy.

[9] World Bank (2017). Georgia Public Expenditure Review: Building a Sustainable Future.

[10] Gale NK, Heath G, Cameron E (2013). Using the framework method for the analysis of qualitative data in multi-disciplinary health research. BMC Med Res Methodol.

[11] (2022). Government of georgia. resolution of the government of georgia on the approval of the national healthcare strategy of georgia for 2022 − 2030. https://matsne.gov.ge/ka/document/view/5453716?publication=0.

[12] National Statistics Office of Georgia Healthcare. https://www.geostat.ge/en/modules/categories/54/healthcare.

[13] OECD (2023). Hospital beds and occupancy. https://www.oecd.org/en/publications/health-at-a-glance-2023_7a7afb35-en/full-report/hospital-beds-and-occupancy_10add5df.html.

[14] Parliament of Georgia (1997). Law of Georgia on medicines and pharmaceutical activities.

[15] Goginashvili K, Nadareishvili M, Habicht T (2021). Can people afford to pay for health care? new evidence on financial protection in georgia. report no.: who/euro:2021-2532-42288-58479. https://apps.who.int/iris/handle/10665/342814.

[16] Galt & Taggart, Curatio International Foundation (2021). Georgian healthcare barometer xiv wave the analysis of financial stability and risks in healthcare | curatio international foundation. http://curatiofoundation.org/georgian-healthcare-barometer-xiv-wave-analysis-financial-stability-risks-healthcare/.

[17] (2021). Pharmaceutical market monitoring report. Georgian competition and consumer protection agency.

[18] Insurance State Supervision Service of Georgia (2023). Financial and statistical indicators of insurance sector: overview of the insurance market 2023. https://insurance.gov.ge/ka/Statistics/GetFile/650?type=4.

[19] Government of Georgia (2013). Resolution No. 36 On measures to be taken during the transition to universal healthcare.

[20] Tvaliashvili M, Sulaberidze L, Goodman C (2024). Exploring the risks of fragmentation in health care markets – an analysis of inpatient care in georgia. social sience and medicine. https://www.sciencedirect.com/science/article/pii/S0277953624008827.

[21] Service for Accounting, Reporting and Auditing Supervision (2022). Annual financial statements. https://saras.gov.ge/en.

[22] Fox JA (2015). Social Accountability: What Does the Evidence Really Say?. World Dev.

[23] WHO Regional Office for Europe (2023). Georgia: moving from policy to actions to strengthen primary health care.

[24] WHO Regional Office for Europe (2021). Rethinking primary health care financing in georgia. https://iris.who.int/bitstream/handle/10665/350562/WHO-EURO-2021-4202-43961-61960-eng.pdf.

[25] UNDP (2022). Assessing public participation in the policymaking process | united nations development programme. https://www.undp.org/georgia/publications/policymaking-public-participation.

[26] Chukwama A, Ghazaryan E, Gong E (2020). Strategic purchasing for better health in armenia (vol. 1 of 2). https://documents.worldbank.org/en/publication/documents-reports/documentdetail/174831600184459793.

[27] Habicht J, Hawkins L, Jakab M Governance for strategic purchasing in kyrgyzstan’s health financing system. https://iris.who.int/server/api/core/bitstreams/17f5d96c-d98b-4dbd-b398-11363148d38d/content.

[28] Moldoisaeva S, Kaliev M, Sydykova A (2022). Kyrgyzstan: Health System Review. Health Syst Transit.

[29] Tangcharoensathien V, Limwattananon S, Patcharanarumol W (2015). Achieving universal health coverage goals in Thailand: the vital role of strategic purchasing. Health Policy Plan.

[30] Marshall AI, Witthayapipopsakul W, Chotchoungchatchai S (2023). Contracting the private health sector in Thailand’s Universal Health Coverage. PLOS Glob Public Health.

[31] Habicht T, Reinap M, Kasekamp K (2018). Estonia: Health System Review. Health Syst Transit.

[32] Habicht T, Habicht J, van Ginneken E (2015). Strategic purchasing reform in Estonia: Reducing inequalities in access while improving care concentration and quality. Health Policy.

