# Supplementary material for: Private provision of health services in Georgia: a qualitative exploration of governance behaviours
Source: BMJ Glob Health. 2025 Dec 17;10(12):e018922. doi: 10.1136/bmjgh-2025-018922 (PMC12716559; doi:10.1136/bmjgh-2025-018922)
Supplement: online supplemental file 1 [file bmjgh-10-12-s001.docx]

Annex 1: List of key informants and workshop participants

| List of key informants |  |
| --- | --- |
| **Group** | **N** |
| Health Policy / Private Health Sector Experts | 2 |
| Ministry of Health / Relevant Department Representatives | 4 |
| Agency for Regulation of Medical and Pharmaceutical Activities | 3 |
| State Purchasing Agency – National Health Agency | 1 |
| Private Medical Facilities | 6 |
| Professional Associations | 2 |
| Competition Agency | 1 |
| Insurance State Supervision Service of Georgia | 1 |
| Donors and International Partners | 1 |
| List of validation workshop participants | |
| **Group** | **N** |
| Ministry of Health / Relevant Department Representatives | 5 |
| Private Medical Facilities | 3 |
| Professional Associations | 1 |
| Donors and International Partners | 1 |

Annex 2 Key sources of information

| Source of information | Web-link |
| --- | --- |
| Government of Georgia. National Health-care Strategy of Georgia 2022–2030. Tbilisi: Government of Georgia; 2022 | <https://matsne.gov.ge/ka/document/view/5453716?publication=0> |
| Government of Georgia. Government resolution on the approval of the procedure for development, monitoring and evaluation of policy documents. Tbilisi: Government of Georgia; 2019 | <https://matsne.gov.ge/ka/document/view/4747283?publication=0> |
| Government of Georgia. Government resolution on the approval of the provisions on the procedure and conditions for issuing a license for medical activity and a permit for an inpatient facility. Tbilisi: Government of Georgia; 2010 | <https://matsne.gov.ge/ka/document/view/3567739?publication=0> |
| Government of Georgia. On Amendments to the Resolution of the Government of Georgia No. 385 of December 17, 2010 "On Approval of the Provisions on the Procedure and Conditions for Issuing Medical Activity Licenses and Permits for Inpatient Institutions". Tbilisi: Government of Georgia; 2017 | <https://matsne.gov.ge/ka/document/view/3567739?publication=0> |
| Government of Georgia. Resolution N 36 on the approval of some of the measures to be taken in order to transition to universal health-care. Tbilisi: 2013. | <https://matsne.gov.ge/ka/document/view/1852448?publication=0> |
| Georgian competition and consumer protection agency. Pharmaceutical market monitoring report. Tbilisi: 2021 | <https://gcca.gov.ge/uploads_script/decisions/tmp/0e920af7373747e089b6921817fb9e12.pdf> |
| State Audit Office. Performance Audit Report: Human Resources for Health. Tbilisi: 2024 | <https://sao.ge/ka/%E1%83%A4%E1%83%A1%E1%83%98%E1%83%A5%E1%83%98%E1%83%99%E1%83%A3%E1%83%A0%E1%83%98-%E1%83%AF%E1%83%90%E1%83%9C%E1%83%9B%E1%83%A0%E1%83%97%E1%83%94%E1%83%9A%E1%83%9D%E1%83%91%E1%83%98%E1%83%A1-%E1%83%90%E1%83%A3%E1%83%93%E1%83%98%E1%83%A2%E1%83%98%E1%83%A120240906034310556ka.html> |
| Asian Development Bank. Support to Health Sector Enhancement Program: Digital Health Assessment. 2023 | <https://www.adb.org/sites/default/files/project-documents/56069/56069-002-tacr-en.pdf> |
| Health information system assessment in Georgia: full assessment report. Geneva: World Health Organization; 2022. |  |
| Health Systems in Action: Georgia | <https://eurohealthobservatory.who.int/publications/i/health-systems-in-action-georgia-2024> |
| Galt and Taggart. Education Sector in Georgia. Tbilisi: 2024 | <https://galtandtaggart.com/en/reports/research-reports/education-sector-georgia> |
| Galt and Taggart. Georgia’s Health-care Sector. Tbilisi: 2023 | [https://api.galtandtaggart.com/sites/default/files/2023-05/report/georgias-health-care-sector-overview_eng_may-23.pdf](https://api.galtandtaggart.com/sites/default/files/2023-05/report/georgias-healthcare-sector-overview_eng_may-23.pdf) |
| National Center for Educational Quality Enhancement. Medical Education in Georgia: Quality Assurance, Main Trends and Challenges. Georgia; 2021 | <https://eqe.ge/res/docs/2020/TA_NCEQE_MEDICINE_ENG_2021.pdf> |
| Goginashvili K, Nadareishvili M, Habicht T. [Can people afford to pay for health-care? New evidence on financial protection in Georgia]. Copenhagen: WHO Regional Office for Europe; 2021 | <https://iris.who.int/handle/10665/342814>. |
| Insurance State Supervision Service of Georgia. 2023. Financial and Statistical Indicators of Insurance Sector: Overview of the Insurance Market 2023. | <https://insurance.gov.ge/ka/Statistics> |
| Service for Accounting, Reporting and Auditing Supervision (SARAS). Annual financial statements. Georgia: 2022 | <https://saras.gov.ge/en> |
| Richardson E, Berdzuli N. Georgia: health system review. Health Systems in Transition. 2017;19(4):1–90 | <https://apps.who.int/iris/handle/10665/330206> |
| Minister of Internally Displaced Persons from the Occupied Territories of Georgia, Labor, Health and Social Protection. Order 41 N "On approval of the procedure for the production of outpatient medical documentation". Tbilisi: 2011 | <https://matsne.gov.ge/ka/document/view/5829454?publication=0> |
| Minister of Internally Displaced Persons from the Occupied Territories of Georgia, Labor, Health and Social Protection. Order 338 N “On approval of the procedure for filling out the health status report and health status report form”. Tbilisi: 2007 | <https://matsne.gov.ge/ka/document/view/5492943?publication=0> |
| Minister of Internally Displaced Persons from the Occupied Territories of Georgia, Labor, Health and Social Protection. On the procedure for producing and providing medical statistical information. Tbilisi: 2019 | <https://matsne.gov.ge/ka/document/view/4509878?publication=0> |
| World Health Organization, Health Financing Progress Matrix assessment: Summary of findings and recommendations. Georgia: 2023 | <https://www.who.int/publications/i/item/9789240081437> |

Annex 3: Framework for Data Collection and Analysis

| **Governance Behaviour** | | **Assessment questions/foci for each GB and, for *Enable Stakeholders*, subdomain/mechanism** |
| --- | --- | --- |
| **Deliver Strategy** | | Do up-to-date documents exist that, individually or collectively, define the government's strategic goals in health? (Note: whether a document can be regarded as 'up-to-date' can be determined by whether the document is still 'in use' in guiding the policy direction of the relevant government entity.) |
|  |  | Do such documents outline clear role(s) for the private health sector in achieving these? |
|  |  | Do such documents outline specific policy mechanisms for achieving such outcomes? |
|  |  | Do they explain how such mechanisms will influence the operation and performance of the private health sector following the identified strategic goals? |
|  |  | Do they identify specific arrangements for implementing them (e.g., by allocating needed financial resources), tracking change, and evaluating the effects of change? |
|  | | |
| **Enable Stakeholders** | *Facility registration and licensing* | Are private facilities legally required to be registered/licensed? |
|  |  | Is the law well-enforced? (E.g. are licensed facilities >50% or >80% of the total number?) |
|  |  | Are licensing conditions well-specified and scaled to the requirements of each service level/ facility type? |
|  |  | Are there a well-defined compliance mechanisms – linked to the risks of each service level/facility type? |
|  |  | Do registration/licensing conditions connect to other regulatory goals – e.g. compliance with observance of clinical guidelines and data reporting rules? |
|  |  | Does the capacity exist in the relevant agency to fully implement registration and licensing processes? (e.g., are inspections undertaken for >50% or >80% of the total number of license applications?) |
|  |  | Are procedures transparent (e.g., do they reduce the potential for bias or corruption in decision-making)? |
|  | *Regulation of clinical practice* | Does a suite of national clinical guidelines, standards, and protocols apply to both public and private sectors (for-profits and non-profits) exist? |
|  |  | Are such guidelines, standards, and protocols evidence-based (e.g., based on a systematic review of the existing scientific literature and/or expert evidence - or some other formal process for ensuring alignment with international best practice)? |
|  |  | Are guidelines, standards, and protocols mandatory in private health facilities? |
|  |  | If the guidelines, standards, and protocols are mandatory, is their application enforced? |
|  |  | Is there a specific entity that is in charge of enforcement mechanisms? |
|  |  | Does the technical capacity exist for effective enforcement, e.g., within the relevant regulatory bodies? |
|  |  | Do outcome measures and performance-based reporting frameworks exist and are implemented? |
|  | *Regulation of retail pharmacy* | Is there a well-defined system for regulating the operation of private pharmacy retailers, including specifications on the presence of a qualified pharmacist for each retail outlet? |
|  |  | Is the related system enforced effectively (i.e., are the numbers of registered pharmacies >50% or >80% of the total number of such retailers)? |
|  |  | Is there an institutional framework for maintaining active registers of all licensed pharmacies? |
|  |  | Are there mechanisms to ensure compliance and enforce defined standards for pharmacies (e.g., sanctions for non-compliance)? |
|  |  | What actions, if any, have been taken to reduce the potential for bias, conflict or corruption in authorities' decisions about licensing? |
|  |  | What actions have been taken to address the availability and use of informal medicine retailers? |
|  | *Regulation of the private health insurance industry* | Are policies in place to safeguard consumers' rights (e.g., guarding against insolvency, fraud, or overly restrictive pay-out clauses)? |
|  |  | Are there policies in place to ensure that (e.g., due to under-insurance) the sickest patients are not being referred to public facilities at a cost to those facilities? |
|  | *Regulation of competition/market structure* | Do state authorities undertake assessments of the competitive situation of the private health sector, either in general or in specific service levels/facility types/services domains (e.g., primary care, outpatient specialist care (or specific specialist services), hospitals, diagnostic services, and pharmacy retail)? |
|  |  | Do state authorities use policy mechanisms to influence the competitive situation of the private health sector in general or specific service domains (e.g., primary care, outpatient specialist care, hospitals, diagnostics, and pharmacy retail)? |
|  |  | Are the extant policy mechanisms effective in preventing the accumulation or abuse of market power (e.g., price or rate-of-return regulation and/or scrutiny of or prevention of mergers and acquisitions)? |
|  | *State purchasing of / contracting with the private sector* | To what extent are private facilities included in publicly financed service delivery (e.g., % of providers contracts (e.g. for inpatient, outpatient, other service areas) with state purchasers is <20%, <50%, >50%, >80)? |
|  |  | To what extent and in what ways do **eligibility criteria**, **contract specifications**, and **payment models** align with equity of access, financial protection, and quality of care goals, alongside the financial sustainability of public spending? *(Note that specific sub-questions will include: (i) Is purchasing selective, criteria-based, or open to all willing providers? (ii) Is balance/extra billing allowed (and, if so, is it regulated or unregulated) or disallowed? (iii) Are prices and service volumes controlled, and in what ways? (iv) Do contracting mechanisms support the ability of small health-care providers to administer contracts effectively? (v) Are payment models retrospective/prospective, and input or output-based?)* |
|  |  | To what extent do monitoring arrangements ensure that equity of access, financial protection and quality of care objectives are met in practice? *(Note that specific sub-questions may include: Are controls on service volumes incorporated in agreements?)* |
|  | | |
| **Foster Relations** | | Has the government established platforms for open, transparent, and purposeful policy dialogue, and do these have a meaningful impact on policy formulation and implementation? |
|  |  | Has the government encouraged the private sector (for-profit and non-profit) to establish representative bodies to engage in purposeful and sustained dialogue? |
|  |  | Have such bodies been established? |
|  |  | How representative are they (e.g., are they inclusive of the full range of ownership types (e.g., sole-proprietor businesses, SMEs, large, limited companies; and facility types (rural/urban clinics, hospitals, etc)? |
|  |  | Has the government taken action to ensure that a broad range of other stakeholders – including patients' associations, community leaders, representatives of vulnerable groups, etc. are included in dialogue structures as a matter of routine? |
|  |  | Has the government taken robust action to eliminate the potential for bias, conflict of interest or corruption in decision-making? |
|  | | |
| **Build Understanding** | | Is there a national HIS? Are private sector entities required to report within the national HIS? What are the incentives and disincentives for doing so (e.g., is reporting mandated as part of licensing)? |
|  |  | To what extent do private sector entities report into the national HIS? Are there concerns with the quality and regularity of reporting (e.g., accuracy, completeness, reliability, relevance, and timeliness)? Are other sources of private sector data/information available and used? (e.g., surveys, assessments, research) |
|  |  | Is the resulting information available in a format that enables all relevant government/health authorities - at the national, regional and local levels - to make evidence-based strategic and operational decisions? |
|  |  | Do relevant government/health authorities systemically use the information to monitor, evaluate and improve policy development and implementation (e.g., through identifying successful pilots of private sector engagement activities that may be considered for scale-up)? |
|  |  | Is any of the data shared with the public to improve its understanding of the operation and performance of the health sector in general or individual entities/providers in particular? |
|  | | |
| **Align Structures** | | Are private sector health entities integrated into health service delivery organisational arrangements (e.g., arrangements account for formal and informal health entities, digital health, and self-care services, etc.)? |
|  |  | Are systems used to align public and private health-care providers towards a primary health-care (PHC)-oriented and nationally defined service delivery model? (e.g., referral, quality assurance, supervision)? |
|  |  | Are structures in place to coordinate the engagement of donors/ development actors with private health-care providers in alignment with the stated roles of the private sector in national health strategies? |
|  |  | Is the private health sector included in all relevant priority health programmes and quality improvement initiatives – e.g., ensuring that reciprocal arrangements are in place to encourage and enable the private sector to contribute to programme goals? |
|  | | |
| **Nurture Trust** | | Do consumer protection laws and social accountability mechanisms exist, and are they sufficiently specified to protect users of the private health sector's services? |
|  |  | Does government act to ensure that such laws and mechanisms are well-enforced and exert meaningful influence on the private health sector's incentives and decision-making, thereby protecting patients' rights, health interests, and general well-being? |
|  |  | Are both sectors (public and private) equally accountable to the stated measures in a way that fosters trust between all health systems actors and between the health system as a whole and the population it serves? |
